# MicroRNA-543 suppresses colorectal cancer growth and metastasis by targeting KRAS, MTA1 and HMGA2

**DOI:** 10.18632/oncotarget.7989

**Published:** 2016-03-08

**Authors:** Chuannan Fan, Yancheng Lin, Yubin Mao, Zhengjie Huang, Allan Yi Liu, Handong Ma, Donghong Yu, Alaiyi Maitikabili, Hongjun Xiao, Chuankai Zhang, Fan Liu, Qi Luo, Gaoliang Ouyang

**Affiliations:** ^1^ State Key Laboratory of Cellular Stress Biology, Innovation Center for Cell Signaling Network, School of Life Sciences, Xiamen University, Xiamen, China; ^2^ Medical College, Xiamen University, Xiamen, China; ^3^ Department of Surgical Oncology, First Affiliated Hospital of Xiamen University, Xiamen, China; ^4^ Engineering Research Centre of Molecular Diagnostics, Ministry of Education, School of Life Sciences, Xiamen University, Xiamen, China

**Keywords:** miR-543, colorectal cancer, metastasis, proliferation, microRNA

## Abstract

miR-543 has been implicated as having a critical role in the development of breast cancer, endometrial cancer and hepatocellular carcinoma. However, the exact clinical significance and biological functions of miR-543 in colorectal cancer (CRC) remain unclear. Here, we found that miR-543 expression significantly downregulated in tumors from patients with CRC, APC^Min^ mice and a mouse model of colitis-associated colon cancer. miR-543 level was inversely correlated with the metastatic status of patients with CRC and the metastatic potential of CRC cell lines. Moreover, ectopic expression of miR-543 inhibited the proliferation and metastasis of CRC cells *in vitro* and *in vivo* by targeting KRAS, MTA1 and HMGA2. Conversely, miR-543 knockdown promoted the proliferation, migration and invasion of CRC cells *in vitro* and augmented tumor growth and metastasis *in vivo*. Furthermore, we found that miR-543 expression was negatively correlated with the levels of KRAS, MTA1 and HMGA2 in clinical samples. Collectively, these data show that miR-543 inhibits the proliferation and metastasis of CRC cells by targeting KRAS, MTA1 and HMGA2. Our study highlights a pivotal role for miR-543 as a suppressor in the regulation of CRC growth and metastasis and suggests that miR-543 may serve as a novel diagnostic and prognostic biomarker for CRC metastasis.

## INTRODUCTION

As one of the most common malignant cancers worldwide, colorectal cancer (CRC) has become the fifth leading cause of cancer death for men and women in China [1]. Distant metastasis accounts for the majority of cancer-related mortality in patients with CRC, and the 5-year survival rate for metastatic CRC is only 10–15% [2–5]. Therefore, a better understanding of the molecular mechanisms involved in CRC metastasis will provide diagnostic and prognostic markers and potential targets for the therapeutic intervention of CRC metastasis. It has been demonstrated that several key molecular events, including mutations in KRAS, aberrant activation of the Wnt and PI3K pathways and TP53 inactivation, are involved in the initiation and progression of CRC [6–9]. In addition to genetic aberrances, epigenetic alterations such as changes in microRNA (miRNA) deregulation and chromatin structure contribute to the development of CRC [10]. miRNAs are endogenous noncoding regulatory RNAs that inhibit gene expression at the post-transcriptional level by binding to the 3′-untranslated-region (3′UTR) of target mRNAs [11, 12]. Although great advances have been achieved in elucidating the specific roles of miRNAs in the initiation, progression and metastasis of CRC, the underlying mechanisms remain largely elusive [5, 10, 13].

As a member of a miRNA cluster located in the imprinted DLK1-DIO3 region on human chromosome 14, miR-543 has been reported to function as a tumor suppressor in breast cancer and endometrial cancer [14, 15], whereas it was found to promote the tumorigenesis of hepatocellular carcinoma [16]. However, none of the previous studies has systematically investigated the functions of the miR-543 in the progression of CRC. In this study, we evaluated the clinical significance and biological functions of miR-543 using clinical CRC samples, tissues from two mouse CRC models, and a panel of CRC cell lines with different metastatic potentials. We demonstrate that miR-543 inhibits the growth and metastasis of CRC cells *in vitro* and *in vivo* by targeting KRAS, MTA1 and HMGA2. Our study suggests that miR-543 may be a critical determinant of CRC progression.

## RESULTS

### miR-543 expression is downregulated in CRC tissues and inversely correlated with CRC metastasis

**Figure 1:**
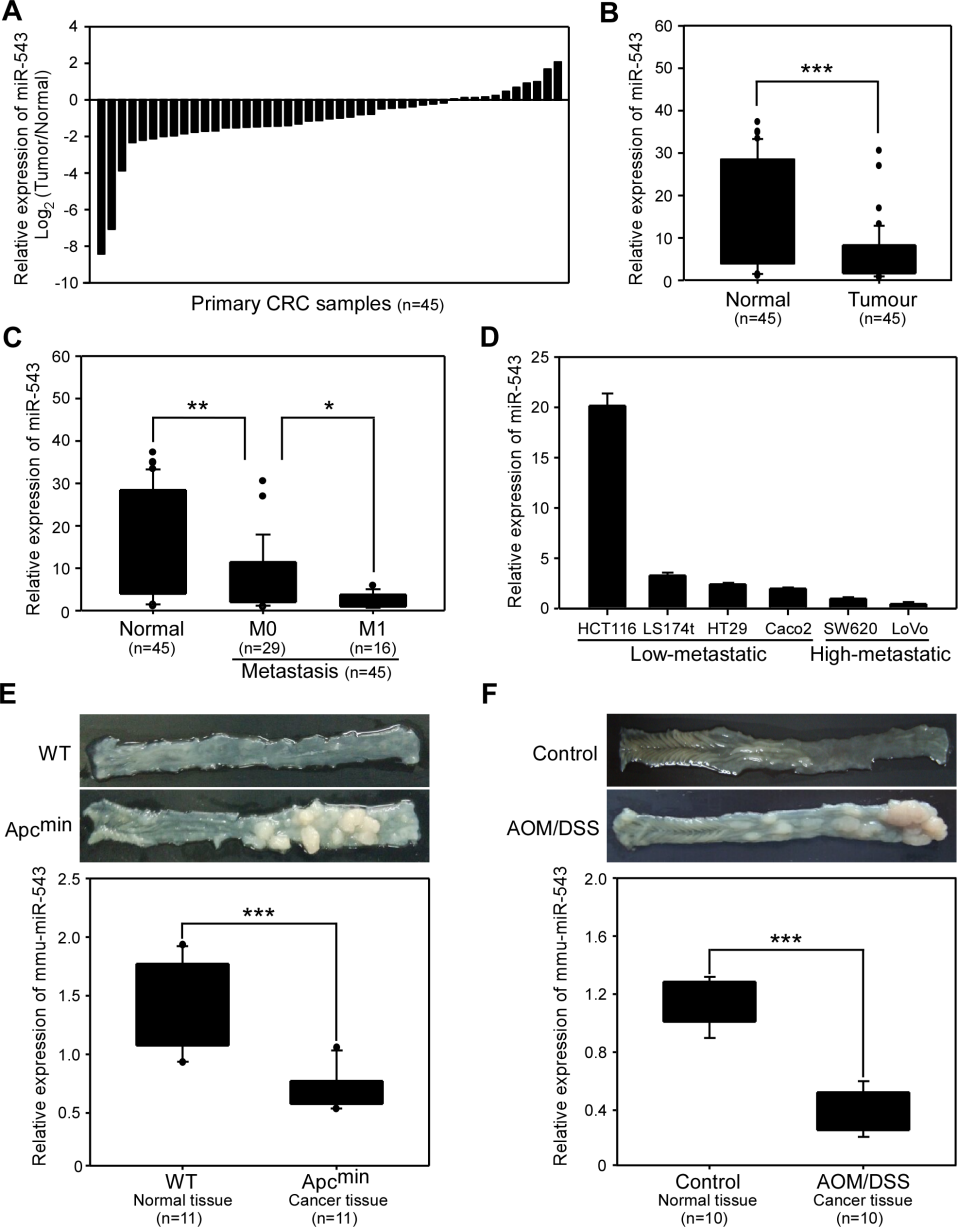
miR-543 expression is downregulated in clinical colorectal cancer (CRC) samples, CRC cell lines and mouse CRC tissues (**A**, **B**) qRT-PCR analysis of miR-543 expression in human CRC tissues and matched normal colon tissues from 45 patients with CRC. Data were expressed as log2 fold change (relative miR-543 expression in tumor sample/relative miR-543 expression in matched normal colon tissue) to show the relative expression in every paired samples (**A**) and the relative expression difference between all normal colon samples and tumor samples (**B**). (**C**) Correlation between miR-543 expression and the distant metastasis status of CRC. (**D**) qRT-PCR analysis of miR-543 expression in CRC cell lines with different metastatic potentials. (**E**, **F**) Representative pictures of colon tissues (top) and qRT-PCR analysis of mmu-miR-543 expression (bottom) in wild-type (WT) and Apc^Min^ mice (*n* = 11) (**E**), and in control and AOM/DSS-treated mice (*n* = 10) (**F**) *p < 0.05, **p < 0.01, ***p < 0.001.

miR-543 has been described as a tumor suppressor gene for breast cancer and endometrial cancer [14, 15] but as an oncogene for hepatocellular carcinoma [16]. To investigate the clinicopathological significance of miR-543 in CRC, we first detected the expression of miR-543 in 45 paired human CRC tissues and matched normal colorectal tissues. As shown in Figure 1A, the level of miR-543 was decreased in 34 of the 45 (75.6%) CRC tissues compared with the normal counterparts. We found that miR-543 expression was reduced by nearly 3-fold in the CRC tissues compared with their corresponding nontumorous colorectal tissues (median 5.8 and 15.7, respectively; *p* < 0.001) (Figure 1B). Clinicopathologic analysis revealed that the expression of miR-543 was also negatively correlated with distant metastasis status (Figure 1C) and N classification (Table 1); however, no significant difference was observed between the level of miR-543 and sex, age or T classification of patients with CRC (Table 1). We further determined the level of miR-543 in highly metastatic human CRC cell lines (SW620 and LoVo) and CRC cell lines with low metastatic potential (HCT116, LS174T, HT29 and Caco-2). The level of miR-543 was relatively lower in highly metastatic CRC cell lines than those in the four tumorigenic but low-metastatic cell lines (Figure 1D), indicating that miR-543 level is inversely correlated with the metastatic potential of CRC cell lines.

**Table 1:** Correlation of relative miR-543 expression with the clinicopathological characteristics of patients with colorectal cancer

Clinicopathological feature	Number of cases	miR-543 levels*		
		High	Low	*P* Value
Age (years)				
> 61	25	15	10	0.12
≤ 61	20	6	14	
Gender				
Male	31	16	15	0.13
Female	14	5	9	
T classification				
I–II	19	7	12	0.082
III–IV	26	8	18	
N classification				
N0	17	12	5	0.009
N1–N2	28	11	17	
Distant metastasis				
No	29	11	18	0.030
Yes	16	4	12	

* The mean (5.8) of the relative expression of miR-543 in tumor tissues of all paired samples was used as a cut-off to classify a tumor sample was High or Low according to their miR-543 expression levels.

To further evaluate the role of miR-543 in CRC progression, we used two mouse CRC models, APC^Min^ mice and azoxymethane/dextran sodium sulfate (AOM/DSS) mice. The APC^Min^ mouse model is a spontaneous CRC model whereas AOM/DSS model is a colitis-associated CRC model [17–19]. Mice in both models formed numerous visible tumors in colorectal epithelium. Using qRT-PCR analysis, we found that the level of miR-543 in CRC tumors isolated from APC^Min^ mice was significantly lower than that in colorectal epithelium tissues from wild-type mice (Figure 1E). Similarly, mice treated with AOM/DSS showed a significantly decreased level of miR-543 in CRC tissues compared with that in colorectal epithelium tissues in the control group (Figure 1F). Taken together, these data demonstrate that miR-543 expression is reduced in clinical CRC specimens and mouse CRC tissues, and its level is inversely correlated with the metastatic potential of CRC cell lines and the metastatic status of patients with CRC.

### KRAS, MTA1 and HMGA2 are direct targets of miR-543

**Figure 2:**
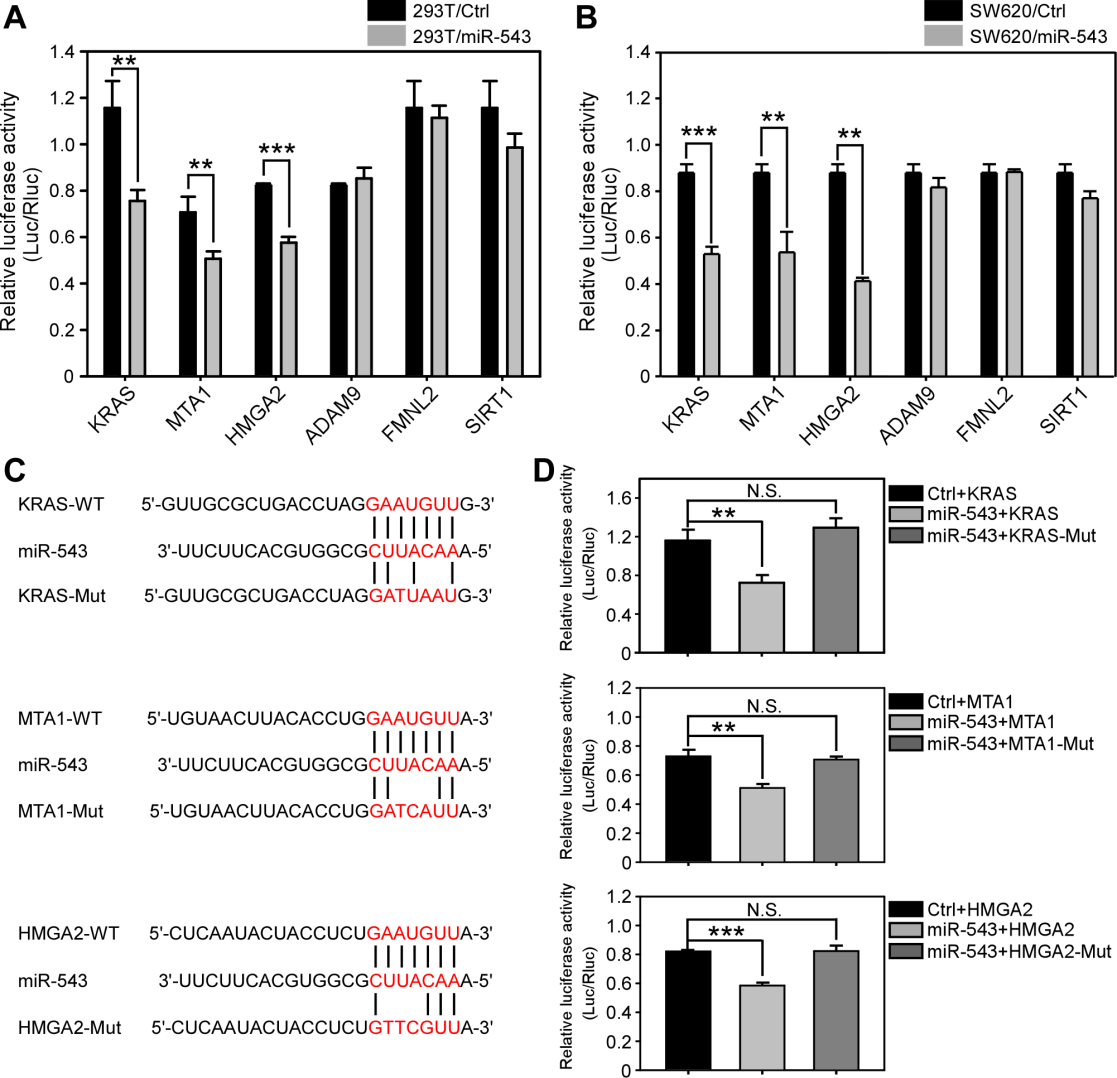
KRAS, MTA1 and HMGA2 are downstream targets of miR-543 (**A**, **B**) Dual luciferase reporter assay analysis of the effects of miR-543 overexpression on the activities of 3′UTRs of predicted target genes in 293T (**A**) and SW620 cells (**B**). (**C**) Mutations were generated in the 3′UTR sequences of the KRAS, MTA1 and HMGA2 mRNAs at the complementary sites for the seed regions in miR-543. (**D**) Dual luciferase reporter assay analysis of the effects of miR-543 expression on the activities of the wild-type and mutant 3′UTRs of KRAS, MTA1 and HMGA2 in 293T cells. These results are representative of at least three independent experiments. **p < 0.01, ***p < 0.001, N.S: no significance.

To explore the tumor-suppressive roles of miR-543 in CRC, we further examined the putative downstream targets of miR-543 by three *in silico* prediction algorithms (miRanda, TargetScan and miRWalk). Several *in silico* prediction algorithm-identified oncogenes including KRAS, MTA1, HMGA2, ADAM9, FMNL2 and SIRT1, which contain putative binding sites for miR-543 in their 3′UTRs, were chosen for further investigation. First, we cloned 3′UTRs that contain putative miR-543 binding sites into the pmiR report luciferase construct, and each was co-transfected with a miR-543 expression plasmid into HEK293T and SW620 cells. Dual-luciferase reporter assays revealed that the luciferase activities of KRAS, MTA1 and HMGA2 but not FMNL2, SIRT1 or ADAM9 significantly decreased in both HEK293T (Figure 2A) and SW620 cells (Figure 2B) upon miR-543 overexpression. However, the inhibitory effects were abolished when the putative miR-543 seed-binding regions in the 3′UTRs of KRAS, MTA1 and HMGA2 were mutated (Figure 2C and 2D). These data demonstrate that KRAS, MTA1 and HMGA2 are direct targets of miR-543.

### miR-543 inhibits CRC cell proliferation *in vitro*

**Figure 3:**
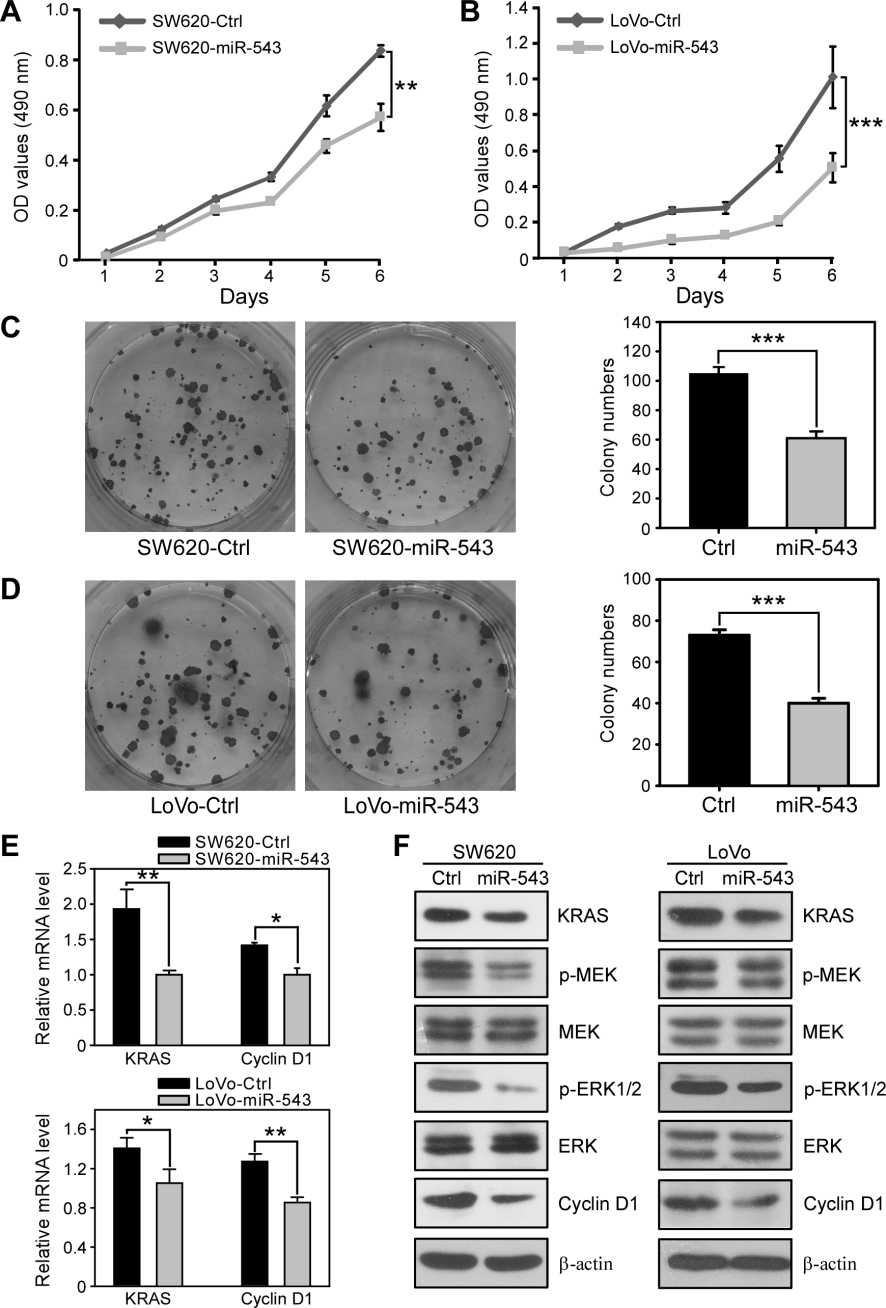
miR-543 overexpression inhibits the proliferation of CRC cells *in vitro* (**A, B**) MTT analysis of the effects of miR-543 on the proliferation of SW620-Ctrl and SW620-miR-543 (**A**) and LoVo-Ctrl and LoVo-miR-543 cells (**B**). (**C**) Representative images and quantification of the colonies formed by SW620-Ctrl and SW620-miR-543 cells. **(D)** Representative images and quantification of colonies formed by LoVo-Ctrl and LoVo-miR-543 cells. (**E**) qRT-PCR analysis of the mRNA levels of KRAS and Cyclin D1 in SW620-Ctrl, SW620-miR-543, LoVo-Ctrl and LoVo-miR-543 cells. (**F**) Western blot analysis of the levels of KRAS and proliferation-related proteins p-MEK, MEK, p-ERK1/2, ERK and Cyclin D1 in SW620-Ctrl, SW620-miR-543, LoVo-Ctrl and LoVo-miR-543 cells. These results are representative of at least three independent experiments. *p < 0.05, **p < 0.01, ***p < 0.001.

To evaluate the role of miR-543 in CRC progression, we first determined the effects of miR-543 overexpression on the biological functions of human CRC cell lines. Two highly metastatic CRC cell lines, SW620 and LoVo, which have very low endogenous miR-543 expression, were stably infected with a lentivirus expressing miR-543 (Supplementary Figure S1). MTT assays revealed that ectopic overexpression of miR-543 in SW620 and LoVo cells resulted in a significant decrease in cell proliferation compared with control cells (Figure 3A and 3B). Moreover, the overexpression of miR-543 significantly decreased the colony numbers of SW620 and LoVo cells (Figure 3C and 3D). The KRAS-RAF-MEK-ERK-Cyclin D1 signaling pathway is a key promoter of the CRC malignant process through its effects on cell proliferation [20, 21]; thus, we next determined whether miR-543 inhibits CRC cell proliferation by targeting KRAS. As shown in Figure 3E, overexpression of miR-543 in SW620 and LoVo cells decreased the expression of KRAS and Cyclin D1 at the mRNA level. Western Blot analysis further revealed that miR-543 overexpression decreased the expression of KRAS and Cyclin D1 and the phosphorylation level of MEK (p-MEK) and ERK (p-ERK) compared with controls (Figure 3F). Together, these results indicate that miR-543 may inhibit the proliferation of CRC cells by downregulating the KRAS-related oncogenic pathway.

### miR-543 suppresses CRC cell migration and invasion *in vitro*

**Figure 4:**
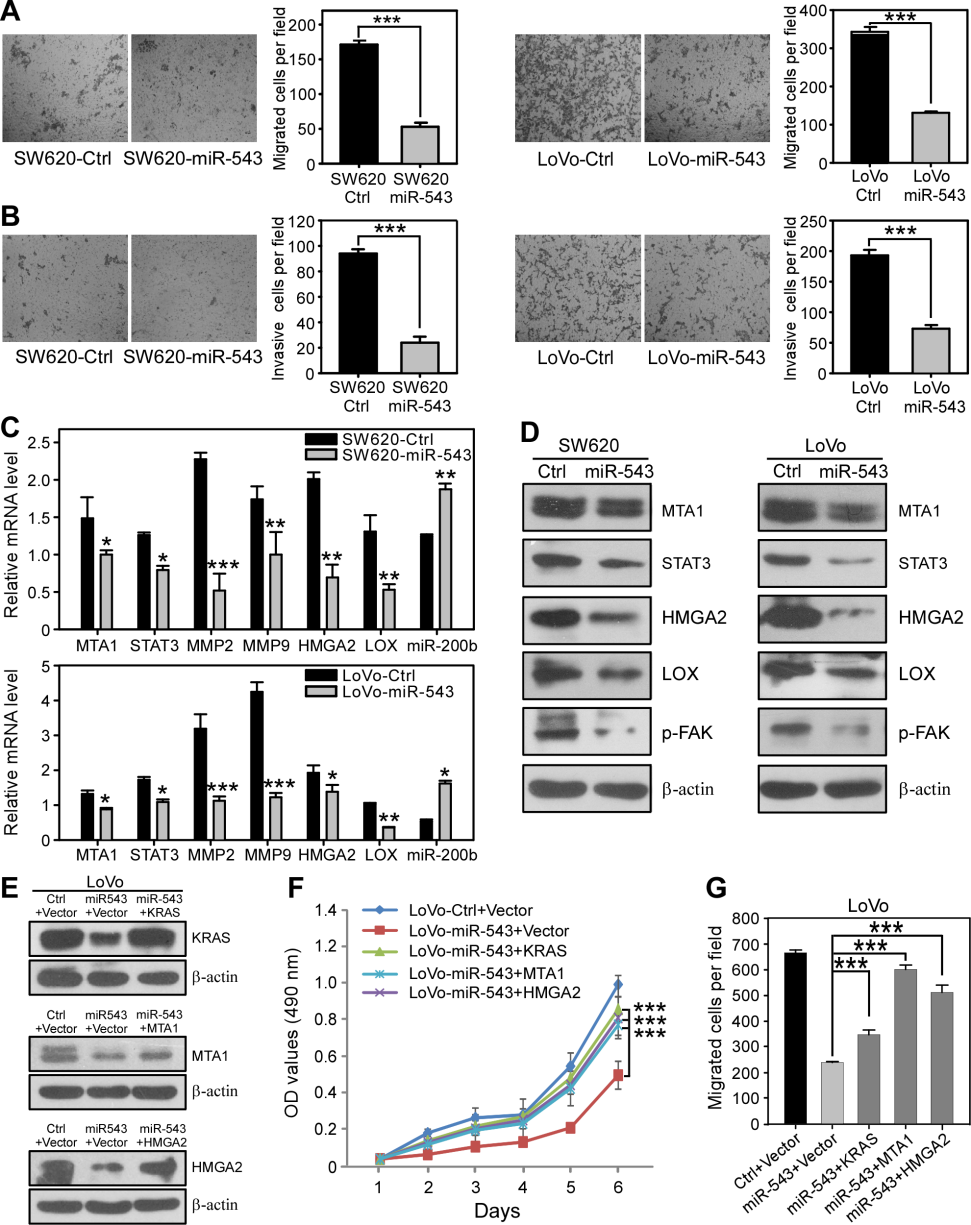
miR-543 overexpression suppresses the migration and invasion of CRC cells *in vitro* and re-expression of KRAS, MTA1 and HMGA2 reverses the miR-543-induced effects on CRC cells (**A**) Transwell migration assays revealed that ectopic overexpression of miR-543 inhibits the migration ability of SW620 and LoVo cells. (**B**) Transwell invasion assay with Matrigel-coated membranes revealed that miR-543 overexpression inhibits the invasion ability of SW620 and LoVo cells. (**C**) qRT-PCR analysis revealed that miR-543 downregulates the mRNA expression of MTA1 and HMGA2 and their downstream genes STAT3, MMP2, MMP9 and LOX but upregulates miR-200b in SW620 (top) and LoVo (bottom) cells. (**D**) Western blot analysis of the effects of miR-543 overexpression on the expression of MTA1 and HMGA2 and their downstream targets STAT3, LOX and p-FAK in SW620 (left) and LoVo (right) cells. (**E**) Western blot analysis of KRAS, MTA1 or HMGA2 re-expression in LoVo-miR-543 cells. LoVo-miR-543 cells were transfected with miRNA-resistant expression constructs for KRAS, MTA1 or HMGA2. Control (Ctrl) represents scrambled miRNA, and vector represents the empty vector used in KRAS, MTA1 and HMGA2 re-expression. (**F, G**) MTT assay (**F**) and migration (**G**) analysis of the proliferation of LoVo-miR-543 cells transfected with miRNA-resistant expression constructs for KRAS, MTA1 or HMGA2. These results are representative of at least three independent experiments. Scale bars, 100 μm. *p < 0.05, **p < 0.01, ***p < 0.001.

We next examined whether miR-543 could suppress the motility and invasiveness of CRC cells by targeting MTA1 and HMGA2. Transwell assays showed that overexpressing of miR-543 in SW620 and LoVo cells significantly inhibited cell migration (Figure 4A) and invasion (Figure 4B) *in vitro*. MTA1 and HMGA2 have been reported to promote EMT and the acquisition of metastatic potential for CRC epithelial cells [22, 23]; thus, we next determined whether the suppression of the migration and invasion of CRC cells by miR-543 was an EMT-related behavior. Upon ectopic overexpression of miR-543, we did not observe significant alterations of the epithelial cell markers E-cadherin and β-catenin or the mesenchymal cell markers N-cadherin and Vimentin in SW620 and LoVo cells (Supplementary Figure S2), indicating that the miR-543-related inhibition of cell migration and invasion is dependent on mechanisms other than EMT. MTA1 has been reported to promote lung metastasis of breast cancer by stimulating STAT3 transcription and the expression of STAT3 target genes [24]. The secretion of MMPs contributes to the abnormalities of the tumor microenvironment during tumor metastasis [25]. We found that miR-543 overexpression in CRC cells decreased the mRNA and protein levels of *MTA1* and *STAT3* and the mRNA level of their downstream genes *MMP2* and *MM*P9 (Figure 4C and 4D). In addition, we also observed that ectopic expression of miR-543 reduced the levels of pro- and cleaved- MMP2 and MMP9 in SW620 and LoVo cells by using gelatin zymography assay (Supplementary Figure S3). HMGA2 can promote breast cancer cell invasion by remodeling the extracellular matrix (ECM) [26]. Here, we found that miR-543 overexpression in SW620 and LoVo cells increased the level of miR-200b but decreased the expression of HMGA2 and LOX at the mRNA (Figure 4C) and protein levels (Figure 4D). Moreover, the phosphorylation level of p-FAK, a downstream effector of LOX [27], was also decreased upon miR-543 overexpression (Figure 4D). These data indicate that miR-543 suppresses CRC invasion *in vitro* by targeting MTA1 and HMGA2.

Next, we performed rescue experiments to further confirm that miR-543 inhibits the malignant phenotypic alterations of CRC cells by directly repressing the three target genes. To abrogate the suppression of miR-543 on KRAS, MTA1 and HMGA2, plasmids expressing each target gene lacking a 3′UTR were constructed and transduced into LoVo-miR-543 cells (Figure 4E). Strikingly, exogenous expression of these three target genes almost completely rescued the miR-543-induced inhibitory effects on cell proliferation (Figure 4F) and colony formation (Supplementary Figure S4). Moreover, restoration of KRAS, MTA1 or HMGA2 in CRC cells at least partially reversed the inhibitory effects on cell migration imposed by miR-543 expression (Figure 4G; Supplementary Figure S5). Therefore, re-expression of KRAS, MTA1 or HMGA2 reverses the miR-543-induced inhibition of the proliferation, migration and invasion of CRC cells *in vitro*, indicating that KRAS, MTA1 and HMGA2 are functional targets of miR-543 in CRC cells.

### miR-543 overexpression inhibits tumor growth and metastasis of CRC cells *in vivo*

**Figure 5:**
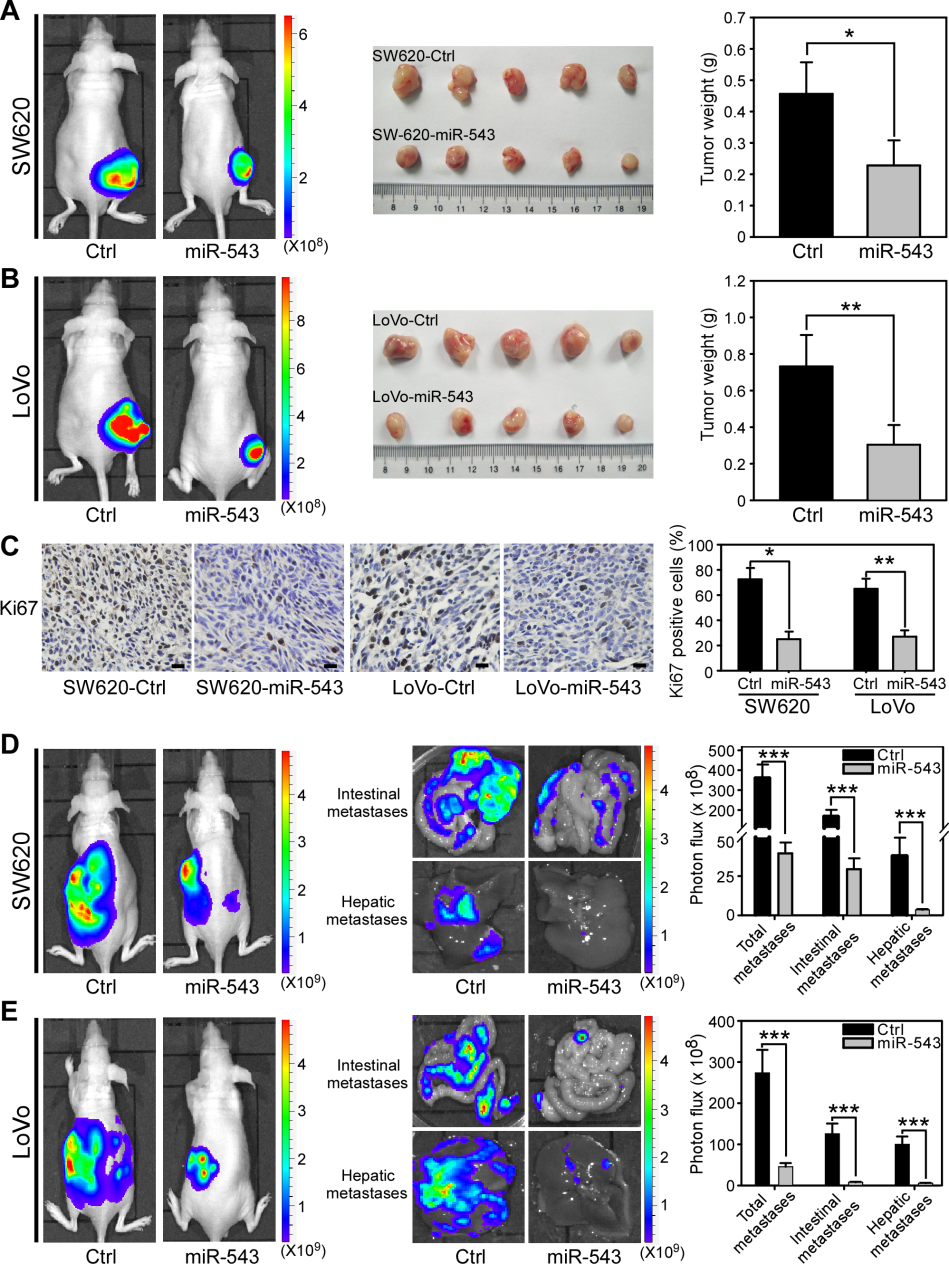
miR-543 overexpression inhibits the tumor growth and metastasis of CRC cells *in vivo* (**A**) Bioluminescence images of nude mice showing tumors derived 28 days after subcutaneous injection in the lower back regions of nude mice with SW620-Ctrl or SW620-miR-543 cells (left). Images of isolated tumors (middle) and quantitation of tumor weights (right) from mice 28 days after injection with SW620-Ctrl or SW620-miR-543 cells. n = 5 per group. (**B**) Bioluminescence images of nude mice showing tumors derived 28 days after subcutaneous injection in the lower back regions of nude mice with LoVo-Ctrl or LoVo-miR-543 cells (left). Images of isolated tumors (middle) and quantitation of tumor weights (right) from mice 28 days after injection with LoVo-Ctrl or LoVo-miR-543 cells. n = 5 per group. (**C**) IHC staining analyses and quantitation of Ki-67-positive tumor cells in xenograft tumors from mice 28 days after injection with SW620-Ctrl, SW620-miR-543, LoVo-Ctrl or LoVo-miR-543 cells. (**D**) Bioluminescence images of metastatic tumors in mice 28 days after intrasplenic injection with SW620-Ctrl or SW620-miR-543 cells (left). Bioluminescence images of intestines and livers isolated from mice 28 days after intrasplenic injection with SW620-Ctrl or SW620-miR-543 cells (middle). Quantitation of metastases in the whole bodies, intestines and livers of these mice (right). n = 5 per group. (**E**) Bioluminescence images of the metastatic tumors in mice 28 days after intrasplenic injection with LoVo-Ctrl or LoVo-miR-543 cells (left). Bioluminescence images of intestines and livers (left) isolated from mice 28 days after intrasplenic injection with LoVo-Ctrl or LoVo-miR-543 cells (middle). Quantitation of metastases in the whole bodies, intestines and livers of these mice (right). n = 5 per group. Scale bars, 50 μm. *p < 0.05, **p < 0.01, ***p < 0.001.

After determining that miR-543 overexpression significantly suppresses the proliferation, migration and invasion of CRC cells *in vitro*, we further investigated whether miR-543 could inhibit the growth and metastasis of CRC cells *in vivo*. SW620 and LoVo cells that stably overexpress miR-543 or their control cells were infected with a virus encoding the luciferase gene and then subcutaneously injected into nude mice and analyzed after 4 weeks. Bioluminescence imaging results showed that mice injected with SW620-Ctrl cells formed larger tumors than those bearing miR-543-overexpressing CRC cells (Figure 5A). We next isolated xenograft tumors and confirmed that the overexpression of miR-543 in SW620 cells significantly decreased subcutaneous tumor growth compared with the control groups, and the volumes and weights of the tumors of the nude mice injected with miR-543-overexpressing cells decreased compared with those of the control group (Figure 5A). Similarly, stable expression of miR-543 inhibited the tumor growth of LoVo cells *in vivo* (Figure 5B). Moreover, tumors from miR-543-overexpressing cells had lower Ki67 expression compared with tumors from controls (Figure 5C). These data indicate that miR-543 inhibits the tumor growth of CRC cells *in vivo*.

Distant spread of CRC cells to the intestines and liver and the formation of macroscopic metastases are the major cause of CRC mortality [28, 29]. We next evaluated whether miR-543 could inhibit the ability of CRC cells to metastasize to the intestines and liver. SW620, LoVo and their miR-543-overexpressing cells, which also expressed the luciferase gene, were intrasplenically injected into nude mice to develop experimental intestinal and hepatic metastasis. As shown in Figure 5D, bioluminescence imaging revealed that miR-543 overexpression significantly reduced the metastasis of SW620 cells in nude mice. We isolated several organs that were reported to have higher CRC metastatic tropism [30] and found that mice bearing miR-543-overexpressing SW620 cells had less intestinal and liver metastases than control mice (Figure 5D; Supplementary Figure S6A). Similar inhibitory effects of miR-543 on tumor metastasis were observed in the mice bearing LoVo-Ctrl or LoVo-miR-543 cells (Figure 5E; Supplementary Figure S6B). Collectively, these observations demonstrate that miR-543 inhibits the tumor growth and metastasis of CRC cells *in vivo*.

### Knockdown of miR-543 promotes the proliferation, invasion and metastasis of CRC cells *in vitro* and *in vivo*

**Figure 6:**
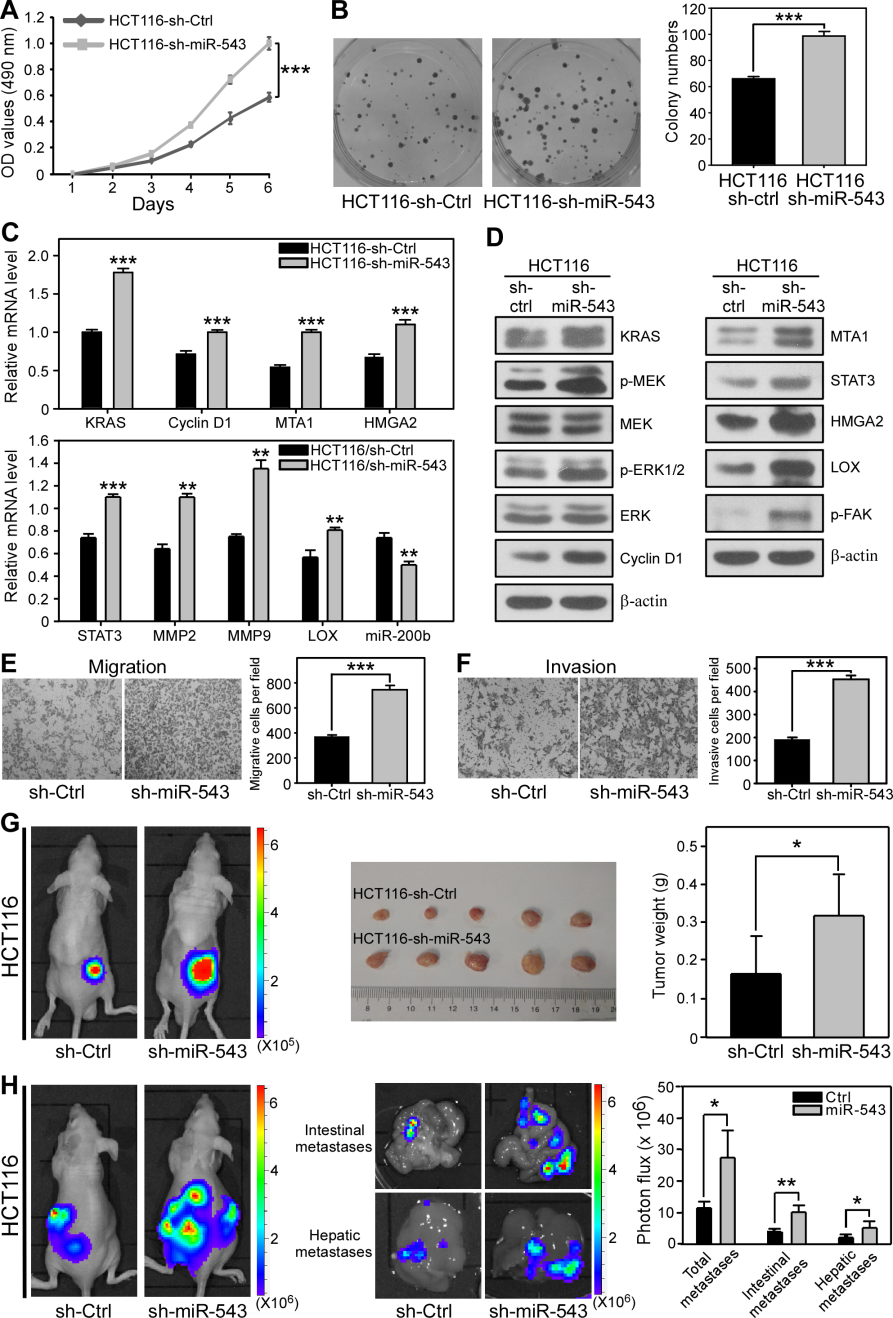
miR-543 knockdown promotes the proliferation, invasion and metastasis of HCT116 cells *in vitro* and *in vivo* . (**A**) MTT analysis of the effects of miR-543 on the proliferation of HCT116 cells. (**B**) Representative images and quantification of colonies formed by HCT116-sh-Ctrl and HCT116-sh-miR-543 cells. (**C**) qRT-PCR analysis revealed that miR-543 knockdown upregulates the mRNA expression of KRAS, MTA1 and HMGA2 and their downstream genes Cyclin D1, STAT3, MMP2, MMP9 and LOX but downregulates miR-200b in HCT116 cells. (**D**) Western blot analysis of the levels of KRAS and the proliferation-related proteins p-MEK, MEK, p-ERK1/2, ERK and Cyclin D1 (left), and MTA1 and HMGA2 and their downstream genes STAT3, LOX and p-FAK (right) in HCT116-sh-Ctrl and HCT116-sh-miR-543 cells. (**E, F**) Transwell migration assays (**E**) and Matrigel-coated membrane Transwell invasion assays (**F**) demonstrated that miR-543 knockdown promotes the migration and invasion of HCT116 cells *in vitro*. (**G**) Bioluminescence images of nude mice showing tumors derived 28 days after subcutaneous injection in the lower back regions of nude mice with HCT116-sh-Ctrl or HCT116-sh-miR-543 cells (left). Images of isolated tumors (middle) and quantitation of tumor weights (right) from mice 28 days after injection with HCT116-sh-Ctrl or HCT116-sh-miR-543 cells. n = 5 per group. (**H**) Bioluminescence images of metastatic tumors in mice 28 days after intrasplenic injection with HCT116-sh-Ctrl or HCT116-sh-miR-543 cells (left). Bioluminescence images of intestines and livers isolated from mice 28 days after intrasplenic injection with HCT116-sh-Ctrl or HCT116-sh-miR-543 cells (middle). Quantitation of metastases in the whole bodies, intestines and livers of these mice (right). n = 5 per group. Scale bars, 200 μm. *p < 0.05, **p < 0.01, ***p < 0.001.

We further performed loss-of-function experiments to verify the function of miR-543 in CRC cells. To this end, we stably knocked down the expression of miR-543 in HCT116 cells, which has relatively high endogenous miR-543 expression, using a lentivirus-based antagomir expression system (Supplementary Figure S7) [31]. As shown in Figure 6A and 6B, miR-543 knockdown led to a significant increase in cell proliferation and colony number. Consistent with these phenotypes, the mRNA levels of KRAS and Cyclin D1 and the protein levels of KRAS, p-MEK, p-ERK and Cyclin D1 upregulated when endogenous miR-543 was suppressed (Figure 6C and 6D). Moreover, in agreement with this result, knockdown of endogenous miR-543 increased the mRNA expression of MTA1, HMGA2, STAT3, MMP2, MMP9 and LOX but decreased the level of miR-200b. The protein levels of MTA1 and HMGA2 and their downstream effectors STAT3, LOX and p-FAK were dramatically upregulated (Figure 6C and 6D). Furthermore, the suppression of endogenous miR-543 dramatically promoted the migration (Figure 6E) and invasion (Figure 6F) of HCT116 cells. However, this pro-invasive phenotype was irrelevant to the EMT program (Supplementary Figure S8). Thus, the loss-of-function data demonstrate that miR-543 knockdown promotes the proliferation, migration and invasion of CRC cells *in vitro*.

We further investigated whether miR-543 knockdown could promote the growth and metastasis of CRC cells *in vivo*. HCT116 cells that stably knockdown miR-543 or their control cells were subcutaneously injected into nude mice for 4 weeks and analyzed by bioluminescence imaging. As shown in Figure 6G, mice injected with HCT116-Ctrl cells formed smaller tumors than those mice bearing miR-543-knockdown CRC cells. Moreover, the metastatic ability of HCT116 cells significantly increased when endogenous miR-543 was knocked down (Figure 6H). These *in vivo* data indicate that knockdown of miR-543 markedly promoted the tumor growth and metastasis of CRC cells.

### Inverse correlation between miR-543 and its targets in clinical CRC samples

**Figure 7:**
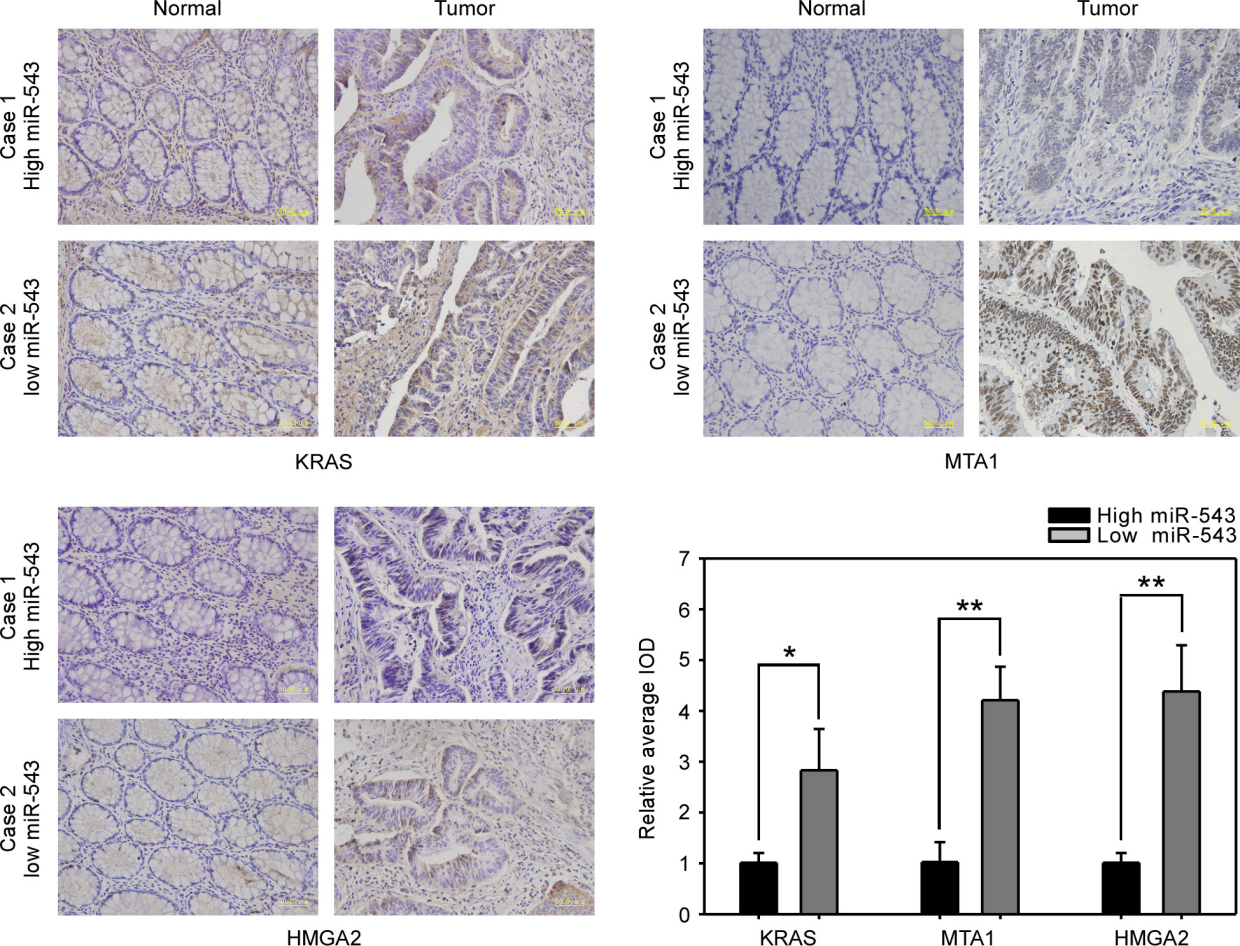
Clinical correlation analysis of the level of miR-543 and its targets in CRC tissues Representative immunohistochemical staining images of the expression of KRAS, MTA1 and HMGA2 in two paired primary CRC tissues with low or high level of miR-543 and analyses of relative average integrated optical density (IOD) using Image Pro-Plus software. Scale bars, 50 μm. *p < 0.05, **p < 0.01.

To extend our findings to human CRC, we examined whether the miR-543-related suppression of KRAS, MTA1 and HMGA2 in CRC cells is clinically relevant. We used two paired CRC samples and their matched nontumorous tissues to detect the protein expression of KRAS, MTA1 and HMGA2 by immunohistochemical staining. Relative miR-543 expression (T/N) was 0.86 in one paired samples and this paired samples was considered to be a case with high miR-543 expression, whereas another paired samples whose relative miR-543 expression was 0.30 was regarded as a case with low miR-543 expression. Quantitative immunohistochemical staining results revealed that CRC tissues with a high level of miR-543 had low expression of KRAS, MTA1 and HMGA2, whereas CRC tissues with a low level of miR-543 exhibited high expression of KRAS, MTA1 and HMGA2, indicating that miR-543 expression is inversely correlated with the levels of KRAS, MTA1 and HMGA2 in clinical CRC tissues (Figure 7).

## DISCUSSION

It has been reported that miR-543 is downregulated in breast cancer [14] and endometrial cancer [15] but functions as an oncogene in hepatocellular carcinoma [16]. In this study, we report that the expression of miR-543 is significantly downregulated in human and mouse CRC tissues and is inversely correlated with the N classification and metastatic status of patients with CRC. Both our *in vitro* and *in vivo* results support that miR-543 significantly inhibits the growth and metastasis of CRC. These findings demonstrate that miR-543 may function as a tumor suppressor or oncogene in a context-dependent manner; therefore, the discrepancy that miR-543 exhibits opposing effects in different tumors deserves further investigation.

Using *in silico* analysis and dual-luciferase reporter assays, we identified KRAS, MTA1 and HMGA2 as three new direct miR-543 downstream targets. KRAS is an oncogene that was found to be mutationally activated in approximately half of early-stage CRCs [32]. MTA1 is a critical regulator of the metastatic process in various types of cancers including CRC [22, 33]. MTA1 enhances lung metastasis of breast cancer by upregulating STAT3 transcription [24], which promotes cell survival, angiogenesis, invasion and metastasis [34, 35]. HMGA2 dysregulation is correlated with poor survival for patients with CRC [36] and promotes breast cancer metastasis by increasing LOX expression [26]. Here, our data reveal that there is an inverse correlation between the miR-543 level and the expression of its targets, KRAS, MTA1 and HMGA2, in clinical CRC samples. Moreover, miR-543 overexpression inhibits the growth and metastasis of CRC cells *in vitro* and *in vivo* by targeting KRAS, MTA1 and HMGA2. Conversely, knockdown of miR-543 promotes the proliferation, invasion and metastasis of CRC cells *in vitro* and *in vivo.* Therefore, these results support the notion that the downregulation of miR-543 in CRC cells “contributes to the activation of multiple oncogenic signaling” pathways by upregulating the expression of KRAS, MTA1 and HMGA2, and ultimately results in CRC progression and metastasis.

Amplifications, deletions or mutations of miRNA loci, epigenetic silencing and transcriptional dysregulation are common mechanisms that lead to the aberrant expression of specific miRNAs [37]. We demonstrated that miR-543 is downregulated in human CRC samples, mouse CRC tissues and CRC cell lines with highly metastatic potential. However, the underlying mechanisms that regulate miR-543 expression remain elusive. In ductal carcinoma samples, CpG islands on the upstream of primary-miR-543 have been shown to be hypermethylated, which directly results in the down-regulation of mature miR-543 [14]. Thus, further investigations are needed to determine whether this epigenetic mechanism or other events are causal factors for the downregulation of miR-543 in CRC progression.

In conclusion, our study highlights a pivotal role for miR-543 as a tumor suppressor in the regulation of CRC cell proliferation and metastasis by targeting KRAS, MTA1 and HMGA2 and suggests that miR-543 may serve as a novel diagnostic and prognostic biomarker for CRC metastasis.

## MATERIALS AND METHODS

### Clinical specimens

For the use of clinical samples for research purposes, prior approval was obtained from the First Affiliated Hospital of Xiamen University. The institutional ethics and scientific committee approved this study. All of the CRC tumor tissues and matched adjacent normal tissues (*n* = 45) from patients with CRC were collected at the Department of Surgical Oncology, First Affiliated Hospital of Xiamen University. The matched clinical information was collected and analyzed with prior written informed consent from the patients. All the tissue biopsies were freshly frozen in liquid nitrogen and stored at −80°C until further use. The medical records of the patients were reviewed to collect the following clinicopathological information: age, gender, metastasis and the clinical stage according to their N classification and T classification. The tumor, node, metastasis (TNM) staging system of the American Joint Committee on Cancer (AJCC) and the International Union Against Cancer (UICC) was used to classify the stage of clinical samples.

### Animal studies

All of the experiments using animals were performed in accordance with a protocol approved by the Animal Care and Use Committee of Xiamen University. Apc^Min^ mice in the C57BL/6 background were provided by Dr Jiahuai Han (Xiamen University, Xiamen). WT and Apc^Min^ mice were euthanized at day 80 after birth. The colitis-associated mouse colon cancer model was induced as follows: on day 1, 6-week-old mice were intraperitoneally injected with 10 mg/kg AOM (Sigma) and maintained on a regular diet and water for 5 days. After 5 days, the mice received water supplemented with 3% DSS (MP Biomedicals) for 5 days. Afterward, the mice were maintained on regular water for 12 days and subjected to two additional DSS treatment cycles. The AOM/DSS group and control mice were euthanized at day 80 after the first injection of AOM. For tumor growth assays, 5 × 10^6^ cells were subcutaneously injected into the lower back regions of 6-week-old male nude mice for four weeks (*n* = 5 per group). For orthotropic metastasis, 6-week-old male nude mice were anesthetized and their spleens were exteriorized by laparotomy, and then 5 × 10^5^ cells were injected into the spleens for four weeks (*n* = 5 per group). Tumor growth and hepatic and intestinal metastases at day 28 were monitored using the live animal Lumina II system (Xenogen IVIS system).

### Cell culture

The human embryonic kidney epithelial cell line 293T and the CRC cell lines SW620 and HCT116 were provided by Dr Han You (State Key Laboratory of Cellular Stress Biology, School of Life Sciences, Xiamen University, Xiamen). LS174t, HT29, Caco2 and LoVo cells were obtained from the Institute of Biochemistry and Cell Biology, Chinese Academy of Sciences, Shanghai. HCT116 cells were maintained in McCoy's 5A media supplemented with 10% fetal bovine serum. 293T, SW620, LS174t and HT29 cells were cultured in DMEM supplemented with 10% fetal bovine serum. Caco2 and LoVo cells were maintained in RPMI1640 media supplemented with 10% fetal bovine serum.

### Plasmid construction and generation of stable cell lines

Plasmid construction was performed as described previously [38]. For the generation of stable miR-543-overexpressing cell lines, a lentivirus-mediated packaging system containing four plasmids—pCDH-miR-543 or control plasmid (scrambled miRNA), pMDL, REV and VSVG—was used. For stable knockdown of miR-543 in HCT116 cells, pLL3.7-puro containing the best hairpin sequence targeting miR-543 (GCGGTGCACTTCTTTTTCA) or a control plasmid (control hairpin) was co-transfected with pMDL, REV and VSVG. The transfection and lentiviral infection processes were similar to those described previously [39]. To generate miRNA-insensitive KRAS, MTA1 and HMGA2 constructs, regions of human KRAS CDS (NM_033360.3) at 193–762 bp, MTA1 CDS (NM_004689.3) at 188–2335 bp and HMGA2 CDS (NM_003483.4) at 812–1141 bp were generated by PCR amplification and then subcloned into the pCMV-5 plasmid (Addgene). The primers used to generate these constructs are listed in Supplementary Table S1.

### Quantitative real-time PCR

Total RNA was prepared with the Trizol reagent (Invitrogen) according to the manufacturer's instructions. For miRNA reverse transcription, cDNA was synthesized using TaqManH MicroRNA Reverse Transcription Kit (ABI) with 100 ng total RNA. For mRNA reverse transcription, cDNA was synthesized using ReverTra AceH qPCR RT Kit (TOYOBO) with 1 mg total RNA. Real-time PCRs were performed using SYBRH Select Master Mix for CFX (Invitrogen). Relative quantification was achieved by normalization to the amount of GAPDH (for mRNAs) or snRNA U6 (for miRNAs). The 2^−ΔΔCt^ (ΔCt = CtmiR-543-CtU6) method for quantitation of gene expression was used to determine miR-543 relative expression levels. The ΔΔCt was calculated by subtracting the ΔCt of the reference sample (a normal clinical or mouse tissue sample was chosen for the normalization of clinical samples or samples in two mouse models, LoVo cell was chosen for the normalization of five CRC cell lines) from the ΔCt of each sample. The mean relative miR-543 expression (5.8) of all clinical tumor samples was chosen as the cut-off to classify a tumor sample was High or Low according to their miR-543 expression level. The primers used are shown in Supplementary Table S2.

### Western blotting

Western blotting was performed as described previously [38]. Cell lysates were subjected to SDS–polyacrylamide gel and immunoblot analysis with antibodies against the following proteins: KRAS and HMGA2 (GeneTex); Vimentin (R & D); Cyclin D1 (Millipore); LOX (Abcam); p-FAK (Invitrogen); E-Cadherin, N-Cadherin and b-catenin (BD); MTA1, STAT3, ERK, p-ERK, MEK and p-MEK (Cell Signaling); b-actin (Sigma Aldrich).

### Cell migration and invasion assays

Migration and invasion assays were performed using Transwell plates (Corning) with 8 μm-pore size membranes without Matrigel (for migration assays) or with Matrigel (for invasion assays). For migration assays, 3 × 10^4^ SW620, 3 × 10^4^ LoVo, 5 × 10^4^ HCT116 cells or the same number of corresponding miR-543-overexpressing or miR-543-knockdown cells were seeded in the top chambers. For the Matrigel-coated membrane Transwell invasion assays, 8 × 10^4^ SW620, 8 × 10^4^ LoVo, 10×10^4^ HCT116 cells or the same number of corresponding miR-543-overexpressing or miR-543-knockdown cells were seeded in the top chambers of Transwell plates. The migrating or invading cells were counted and photographed.

### miRNA reporter luciferase assay

293T or SW620 cells were seeded into a 24-well plate and cotransfected with miR-543 or control and 3′UTR-luciferase plasmids. The cells were lysed at 48 h posttransfection, and the luciferase activity was measured using the Dual-Glo Luciferase Assay System (Promega) and normalized to Renilla luciferase activity.

### Cell proliferation and colony formation assay

For cell proliferation assay, cells were seeded on 96-well plates at initial density of (3 × 10^3^ cells/well). The cells were stained with 100 μl sterile MTT dye (0.5 mg/ml, Sigma-Aldrich, MO) at each time point for 4 h at 37°C followed by removal of the culture medium and addition of 150 μl of dimethyl sulphoxide. The absorbance was measured at 490 nm. All experiments were performed in triplicates. For colony formation assay, cells were seeded in six-well plates at a density of 300 cells/well and maintained in medium for 12–20 days. The cells were washed with PBS, fixed in methanol for 15 min and stained with crystal violet for 15 min. The plates were then photographed, and the colonies were counted. At least three independent experiments were carried out for each assay.

### Gelatin zymography

Cells were plated onto 6 cm culture plates and were incubated with medium that did not contain FBS at 37°C. After 24 h, the medium collected from the incubated cells was concentrated and mixed with substrate gel sample buffer and then loaded without boiling onto 8% SDS polyacrylamide gel that contained 1% gelatin (Sigma, St. Louis, MO). After performing electrophoresis, the gel was then soaked in 2% Triton X-100 with gentle shaking for 60 min with single change of detergent solution. The gel was rinsed and next incubated for 1 day in substrate buffer (50 mM Tris–HCl, pH 7.5, 5 mM CaCl_2_, and 0.02% NaN_3_). Following the incubation, the gel was stained with 0.05% Coomassie brilliant blue G-250 and then destained in 10% acetic acid and 20% methanol.

### Immunohistochemical staining

Tissue sections from specimens of primary colorectal cancer were fixed in 10% formaldehyde and embedded in paraffin. Sections were then cut and stained using immunohistochemistry as previously described [39]. Briefly, paraffin-embedded tissue sections were processed for antigen retrieval by heating the sections in 10 mM sodium citrate (pH 6.0) at 95°C for 20 min. Sections were immunostained with antibodies anti-KRAS (1:200), anti-MTA1 (1:200) or anti-HMGA2 (1:200). The immunostaining was performed with an ABC staining system (Santa Cruz Biotechnology, Santa Cruz, CA) using an avidin-biotinylated-peroxidase detection method. For quantitation, average integrated optical density (IOD) was obtained by analyzing five fields in each slide evaluated by Image-Pro Plus software (version 6.0) for immunohistochemical staining of KRAS, MTA1 and HMGA2. The average IOD of tumor tissue was divided by the average IOD of paired normal tissue to get the relative average IOD.

### Statistical analysis

All quantitative data are expressed as the mean ± SD. Significant differences were analyzed using Student's *t*-test to compare two groups of independent samples. *p* values of 0.05 or less were considered as significant.

## SUPPLEMENTARY MATERIALS FIGURES AND TABLES


