# Supplementary material for: MicroRNA-543 suppresses colorectal cancer growth and metastasis by targeting KRAS, MTA1 and HMGA2
Source: Oncotarget. 2016 Mar 8;7(16):21825–39. doi: 10.18632/oncotarget.7989 (PMC5008326; doi:10.18632/oncotarget.7989)
Supplement: Supplementary file 1 [file oncotarget-07-21825-s001.pdf]

# MicroRNA-543 suppresses colorectal cancer growth and metastasis by targeting KRAS, MTA1 and HMGA2

## Supplementary Materials

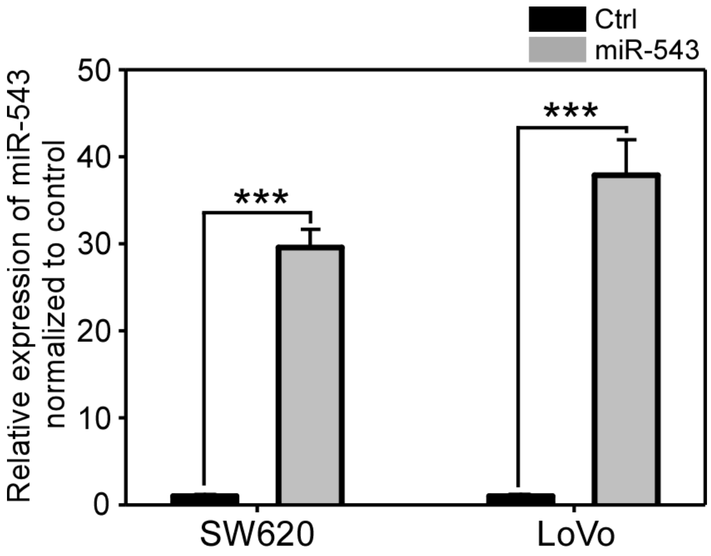


**Supplementary Figure S1: qRT-PCR analysis of miR-543 levels after the ectopic expression of miR-543 in SW620 and LoVo cells.** ***$p < 0.001$.

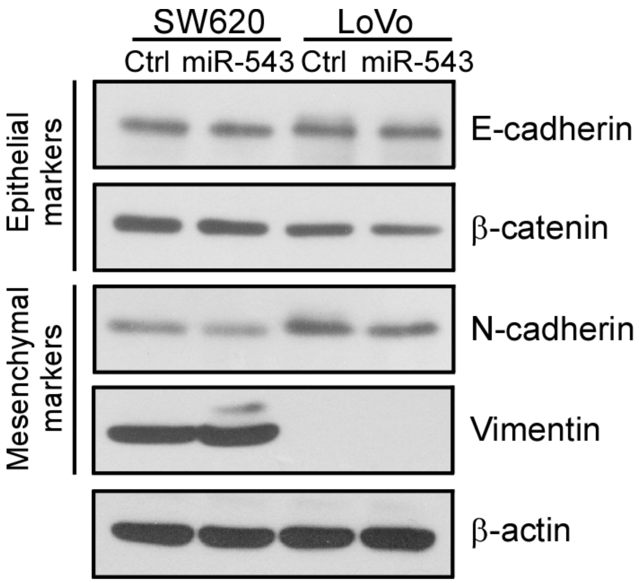


**Supplementary Figure S2: The effects of ectopic expression of miR-543 on the expression of EMT markers in SW620 and LoVo cells were determined by western blotting.**

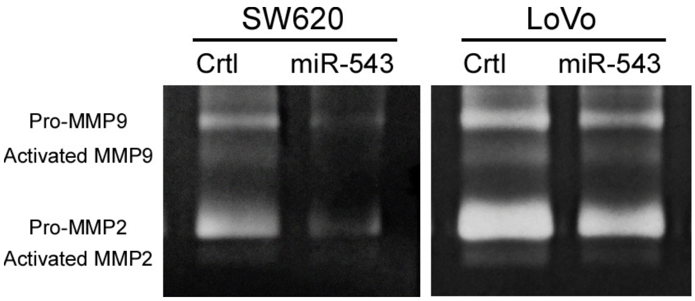


**Supplementary Figure S3: miR-543 downregulates MMP-2 and MMP-9 activities in SW620 and LoVo cells as shown by gelatin zymograph assay.**

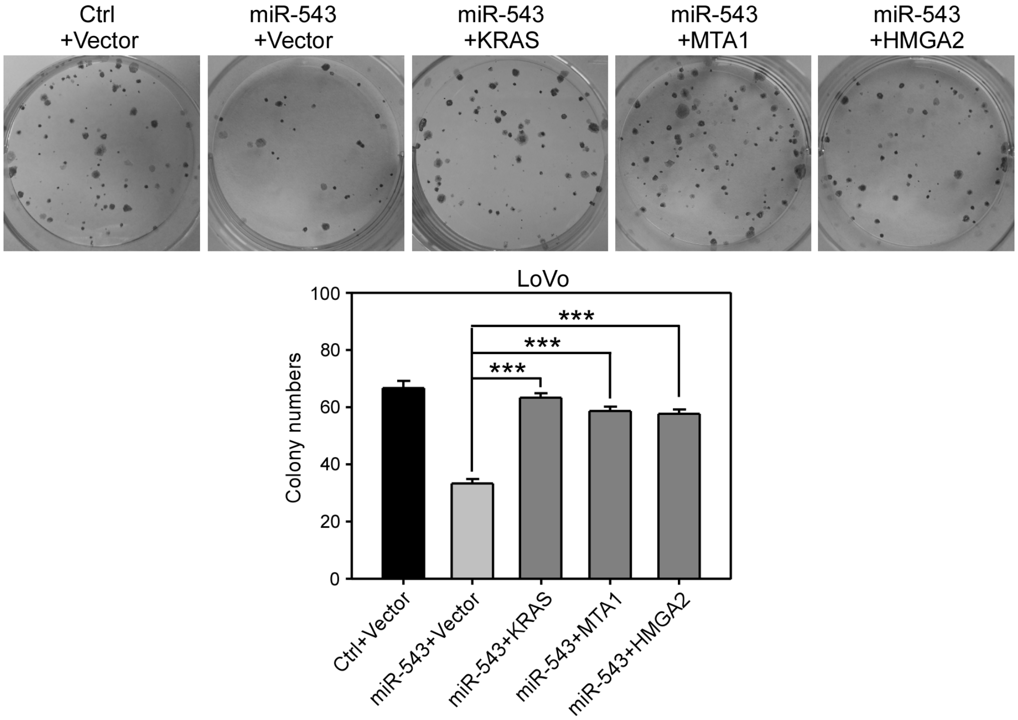


**Supplementary Figure S4: Re-expression of KRAS, MTA1 and HMGA2 reverses the miR-543-dependent inhibitory effects on the colony formation of LoVo cells.** LoVo-miR-543 cells were transfected with miRNA-resistant expression constructs encoding KRAS, MTA1 or HMGA2. Control (Ctrl) represents the scrambled miRNA, and vector represents the empty vector used for KRAS, MTA1 and HMGA2 re-expression. ***$p < 0.001$.

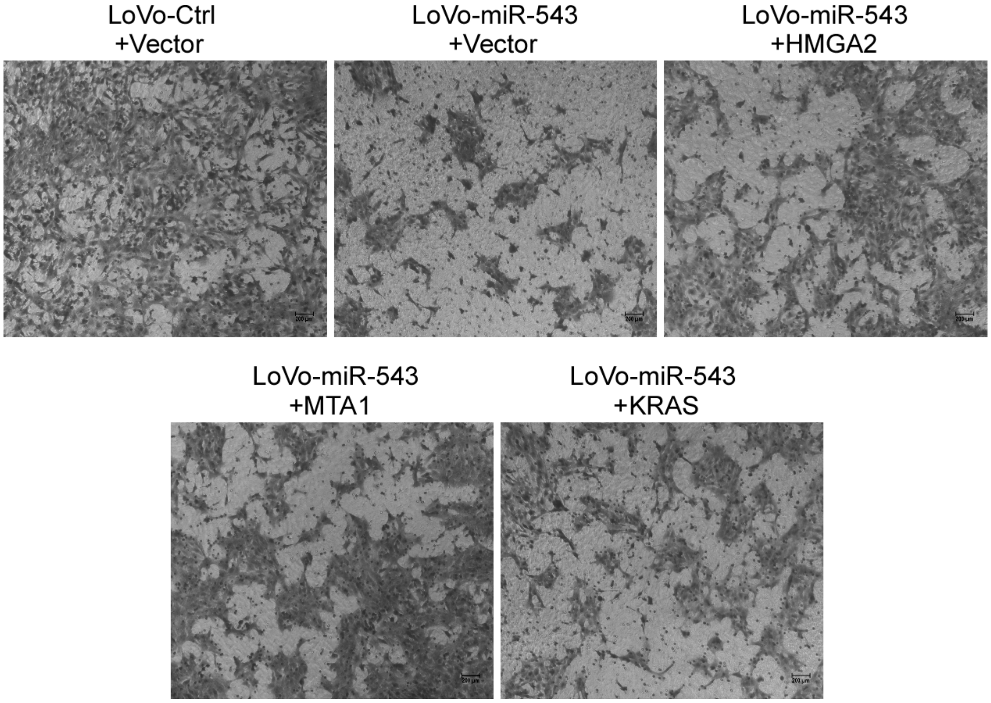


**Supplementary Figure S5: Cell migration analysis of the re-expression of KRAS, MTA1 or HMGA2 in LoVo-miR-543 cells.**

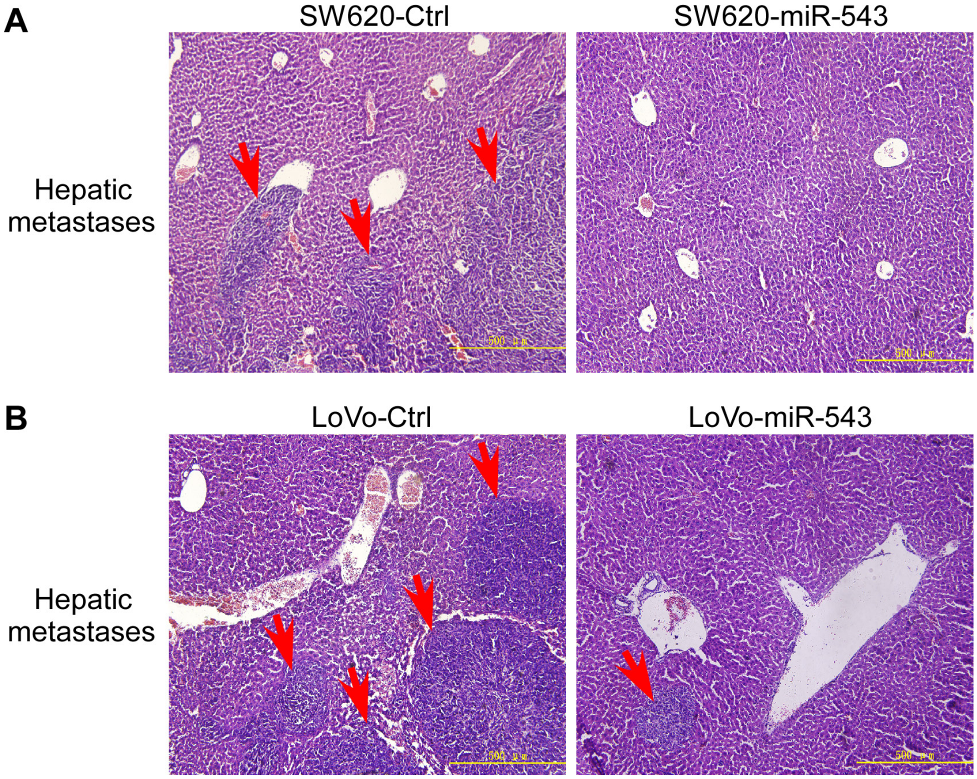


**Supplementary Figure S6: Representative H & E staining images of hepatic metastases in mice intrasplenically injected with SW620-Ctrl, SW620-miR-543 (A), LoVo-Ctrl or LoVo-miR-543 (B) cells for 4 weeks.**

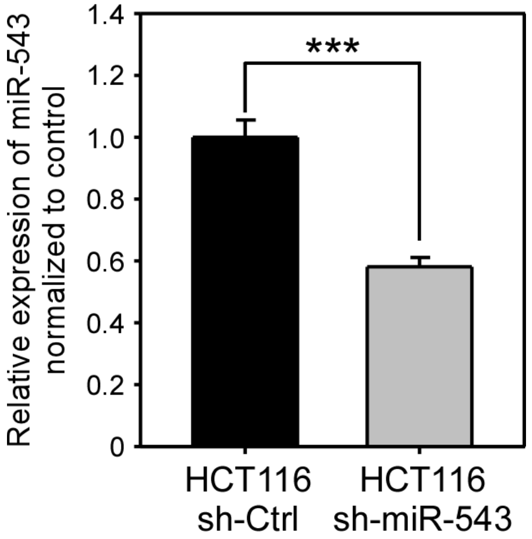


**Supplementary Figure S7: qRT-PCR analysis of the miR-543 knockdown efficiency in HCT116 cells.** ***$p < 0.001$.

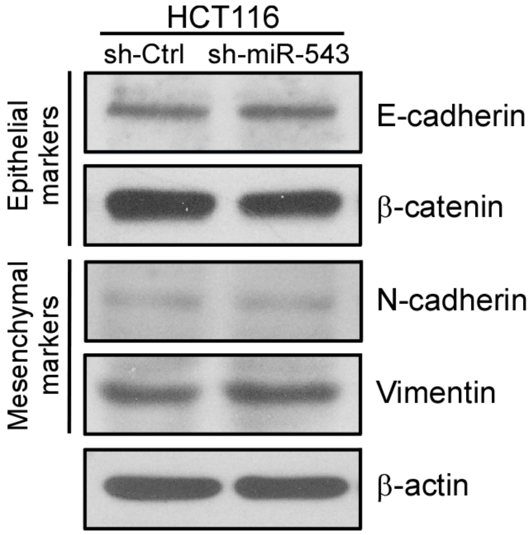


**Supplementary Figure S8: The effects of miR-543 knockdown on the expression of EMT markers in HCT116 cells were determined by western blotting.**

**Supplementary Table S1: Sequences of plasmid constructs**

| | | |
|---|---|---|
| Hsa-miR-543 | Fw<br>Rv | GGGCAGACCTGTTAGATGTCTCC<br>GGGTCAATGCAATCAAAGCACAC |
| KRAS 3′UTR | Fw<br>Rv | GAAGTGCCTGTTTGGGATAATGATAG<br>GATCACTTCACAGCACATACTCCTA |
| MTA1 3′UTR | Fw<br>Rv | CTTATTCCGAGAATGCCGAGGAG<br>ACTTGAAAAGACAAGGGCCAACC |
| HMGA2 3′UTR | Fw<br>Rv | GAGTCTGCCGAAGAGGACTAG<br>AATGACTTGTGAGTGTCTCCC |
| ADAM9 3′UTR | Fw<br>Rv | TCAGGGAACTGAGCTAATAC<br>CTTGATTGTTAAAATAACATGCTGTC |
| FMNL2 3′UTR | Fw<br>Rv | CAAGTGCCTAAAAATGGAAGTACCTG<br>TGC TCATATGCCA TGAAAAGACC AG |
| SIRT1 3′UTR | Fw<br>Rv | AATTGTGCAGGTACAGGAATTGTTC<br>GCACACA AACATCATGC AAATGG |
| KRAS 3′UTR mutation | Fw<br>Rv | TGACCTAGGATACTTGGTCATATCAAACAT<br>ATATGACCAAGTATCCTAGGTCAGCGCAACCA |
| MTA1 3′UTR mutation | Fw<br>Rv | TACACCTGGATACTTAGGATCGTGCGGCCG<br>ACGATCCTAAGTATCCAGGTGTAAGTTACA |
| HMGA2 3′UTR mutation | Fw<br>Rv | CTACCTCTGATACTTACAACGAATTTACAG<br>TTCGTTGTAAGTATCAGAGGTAGTATTGAG |
| sh-miR-543 | Fw<br>Rv | TGCGGTGCACTTCTTTTTCATTCAAGAGATGAAAAAGAAGTGCACCGCTTTTTTC<br>TCGAGAAAAAAGCGGTGCACTTCTTTTTCATCTCTTGAATGAAAAAGAAGTGCAC-CGCA |
| KRAS CDS | Fw<br>Rv | ACTGAATATAAACTTGTGGTAGTTGGAGC<br>TCATCAACACCCAGATTACATTATAATGC |
| MTA1 CDS | Fw<br>Rv | GCCGCCAACATGTACAGGGT<br>CTAGTCCTCGATGACGATGGGC |
| HMGA2 CDS | Fw<br>Rv | AGCGCACGCGGTGAGGGCG<br>CTAGTCCTCTTCGGCAGACTCTTGTG |

**Supplementary Table S2: Primer sequences for qRT-PCR analyses**

| | | |
|---|---|---|
| miR-543 reverse | Reverse primer | GTCGTATCCAGTGCGTGTCGTGGAGTCGGC AATTGCACTGGATACGACTAAGAAG |
| miR-543 Real-time | Fw<br>Rv | GGGGAAACATTCGCGGTGCA<br>TGCGTGTCGTGGAGTC |
| miR-200b reverse | Reverse primer | GTCGTATCCAGTGCGTGTCGTGGAGTCGGC AATTGCACTGGATACGACTACATCA |
| miR-200b Real-time | Fw<br>Rv | GGGGTAATACTGCCTGGTAA<br>TGCGTGTCGTGGAGTC |
| U6-reverse | Reverse primer | CGCTTCACGAATTTGCGTGTCAT |
| U6-real-time | Fw<br>Rv | GCTTCGGCAGCACATATACTAAAAT<br>CGCTTCACGAATTTGCGTGTCAT |
| KRAS | Fw<br>Rv | GTAGGCAAGAGTGCCTTGACG<br>GTACTCCTCTTGACCTGCTGTG |
| MTA1 | Fw<br>Rv | CAGACAAGCAGATCGACGAGTT<br>TGGCGTGCAA CAGGGTGAT |
| HMGA2 | Fw<br>Rv | CAAGTTGTTCAGAAGAAGCCTGC<br>CATGGCAATA CAGAATAAGT GGTCAC |
| Cyclin D1 | Fw<br>Rv | CCGTCCATGCGGAAGATC<br>ATGGCCAGCGGGAAGAC |
| LOX | Fw<br>Rv | GCATACAGGGCAGATGTCAGA<br>TTGGCATCAAGCAGGTCATAG |
| STAT3 | Fw<br>Rv | GGCATTCGGGAAGTATTGTCG<br>GGTAGGCGCCTCAGTCGTATC |
| MMP2 | Fw<br>Rv | GCCCCAGACAGGTGATCTTG<br>GCTTGCGAGGGAAGAAGTTGT |
| MMP9 | Fw<br>Rv | AGACGGGTATCCCTTCGACG<br>AAACCGAGTTGGAACCACGAC |
| GADPH | Fw<br>Rv | GGTCTCCTCTGACTTCAACA<br>GTGAGGGTCTCTCTCTTCCT |
